# Primär sklerosierende Cholangitis – Diagnose und Therapie 2024

**DOI:** 10.1007/s00108-024-01697-0

**Published:** 2024-03-18

**Authors:** Michael Trauner, Emina Halilbasic, Elisabeth Tatscher, Peter Fickert

**Affiliations:** 1 Klinische Abteilung für Gastroenterologie und Hepatologie, Universitätsklinik für Innere Medizin III, Medizinische Universität Wien, Währinger Gürtel 18–20, 1090 Wien, Österreich; 2 Klinische Abteilung für Gastroenterologie und Hepatologie, Universitätsklinik für Innere Medizin, Medizinische Universität Graz, Auenbruggerplatz 15, 8036 Graz, Österreich

**Keywords:** Cholestase, Chronisch-entzündliche Darmerkrankung, Cholangiozelluläres Karzinom, Endoskopische retrograde Cholangiopankreatikographie, Ursodesoxycholsäure, Cholestasis, Chronic inflammatory bowel disease, Cholangiocellular carcinoma, Endoscopic retrograde cholangiopancreatography, Ursodeoxycholic acid

## Abstract

Die Ursache der primär sklerosierenden Cholangitis (PSC) bleibt unklar und erklärt das Fehlen einer kausalen Therapie. Die differenzialdiagnostische Abgrenzung zur noch selteneren IgG4-assoziierten Cholangitis (IAC) gelingt uns immer besser. Fortschritte im Wissen um unterschiedliche klinische Verläufe, Verbesserungen in der nichtinvasiven Diagnostik durch moderne Magnetresonanzbildgebung und die Einführung der Leberelastographie führten zur Entwicklung verbesserter Prognosemodelle. Die Evidenz für Empfehlungen zur medikamentösen (z. B. Ursodesoxycholsäure) oder endoskopischen Therapie (z. B. Ballondilatation und/oder Stenteinlage) bei PSC bleibt gering. Hingegen werden die Langzeitergebnisse der Lebertransplantation bei PSC stetig besser. Mangels hochsensitiver und spezifischer Screeningmethoden gelingt die Früherkennung des cholangiozellulären Karzinoms (CCC) als wichtigste Komplikation selten. Die stetige Verbesserung von ERCP und direkter Cholangioskopie in Kombination mit molekularbiologischen und FISH-Analysen der gewonnenen Gewebsproben ist für die verfeinerte Diagnostik vielversprechend. Aufgrund des deutlich erhöhten Risikos für kolorektale Karzinome wird bei Vorliegen einer chronisch-entzündlichen Darmerkrankung (CED) die jährliche Koloskopie empfohlen. Errungenschaften in der Frühdiagnostik und die erfolgreiche Testung neuer Therapiemodalitäten lassen auf eine stetige Verbesserung in der Betreuung dieser komplexen PatientInnen hoffen.

## Abstract (en)

The etiology of primary sclerosing cholangitis (PSC) remains unclear, which explains in part the lack of a causal treatment. The differential diagnostic distinction from the even rarer immunoglobulin 4 (IgG4)-associated cholangitis (IAC) is becoming increasingly more successful. Advances in the understanding of different clinical courses, improvements in noninvasive diagnostics through modern magnetic resonance imaging (MRI) and the introduction of liver elastography have led to the development of improved prognostic models. The evidence for recommendations on medicinal (e.g., ursodeoxycholic acid) or endoscopic treatment (e.g., balloon dilatation and/or stent insertion) for PSC is still low. In contrast, the long-term results of liver transplantation in PSC patients are constantly improving. Due to the lack of highly sensitive and specific screening methods the early recognition of cholangiocellular carcinoma (CCC) as the most important complication is rarely successful. The continuous improvement of endoscopic retrograde cholangiopancreatography (ERCP) and direct cholangioscopy in combination with molecular biological and fluorescence in situ hybridization (FISH) analyses of bile duct tissue samples are promising for refined diagnostics. Due to the significantly increased risk of colorectal cancer, an annual colonoscopy is recommended in the presence of inflammatory bowel disease. Improvement of the early diagnostics of PSC and successful testing of new treatment strategies raise hope for a continuous improvement in the medical support of these complex patients.

## Einleitung

Die möglichen Ursachen einer sklerosierenden Cholangitis mit periduktaler Fibrose sind vielfältig. Kann keine dieser sekundären Ursachen gefunden werden, so sprechen wir von einer primär sklerosierenden Cholangitis (PSC), deren Diagnose und Therapie auch 2024 eine klinische Herausforderung bleibt. Die Ursache der PSC ist nach wie vor unklar und erklärt das Fehlen einer kausalen Therapie [1–3]. Die Assoziation mit einer chronisch-entzündlichen Darmerkrankung (CED) weist auf eine mögliche pathogenetische Rolle der Darm-Leber-Achse hin. Die Evidenz der Empfehlungen zur medikamentösen oder endoskopischen Therapie ist gering. Trotz ihrer Seltenheit stellt die PSC, gemeinsam mit anderen immunmediierten Lebererkrankungen, eine der häufigsten Transplantationsindikationen dar. Annähernd 50 % der Todesfälle bei PSC sind nicht den Folgen einer Leberzirrhose, sondern Malignomen (insbesondere cholangiozellulären und kolorektalen Karzinomen) zuzuschreiben. Die Frühdiagnose des cholangiozellulären Karzinoms (CCC) ist äußerst herausfordernd, die stetige Verbesserung der Cholangioskopie in Kombination mit erweiterten Analysen der gewonnen Gewebsproben erscheint hier vielversprechend. Zu den Überwachungsstrategien das CCC gibt es leider keine wesentlichen Fortschritte, für kolorektale Karzinome (CRC) wird bei Vorliegen einer CED die jährliche Koloskopie empfohlen. Ziel dieser Übersicht ist, eine Hilfestellung zur klinischen Entscheidungsfindung im Management bei PSC zu geben.

## Diagnose und Differenzialdiagnose

**Figure 1:**
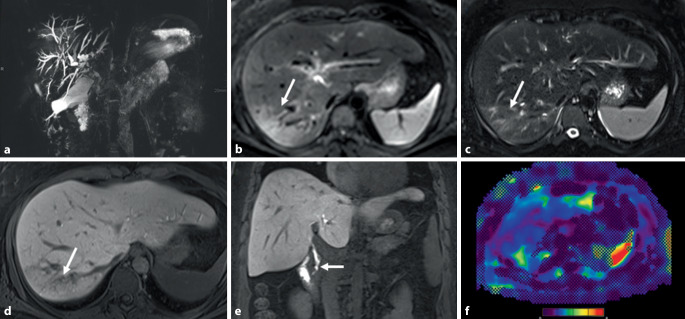

Bei anhaltender Erhöhung der cholestatischen Leberenzyme wie der alkalischen Phosphatase (AP) sollte differenzialdiagnostisch nach Ausschluss einer PBC oder durch Steine oder Tumoren verursachten Cholestase an eine PSC gedacht werden [4]. Die meisten PatientInnen sind asymptomatisch, können sich jedoch mit Bauchschmerzen im rechten oberen Quadranten, Ikterus und/oder Juckreiz, gelegentlich sogar mit Symptomen einer akuten Cholangitis wie Bauchschmerzen, Fieber und/oder Ikterus präsentieren. Die PSC geht häufig (50–80 %) mit einer CED einher, das Vorliegen einer solchen kann daher richtungsweisend in der Diagnostik sein. Die Magnetresonanzcholangiopankreatikographie (MRCP) stellt den Goldstandard in der Diagnostik der PSC dar [4]. Mit einer Sensitivität von 86 % und einer Spezifität von 94 % hat die MRCP als nichtinvasive, risikoarme, nicht strahlenbelastende sowie kostengünstigere Untersuchung die endoskopische retrograde Cholangiopankreatikographie (ERCP) als primäres diagnostisches Verfahren abgelöst. Die charakteristischen, aber unspezifischen Merkmale der PSC sind multifokale Strikturen und Dilatationen der intra- und/oder extrahepatischen Gallengänge (Abb. 1). Weitere Befunde umfassen duktale Wandverdickungen und Gallengangsverlust. Zudem liefert die Durchführung einer umfassenden, qualitativ hochwertigen MRT-Untersuchung mit Verwendung leberspezifischer Kontrastmittel hilfreiche Informationen zu Wanddicke und Schädigung von Gallengängen, Beschaffenheit des Leberparenchyms und möglichen Komplikationen einer fortgeschrittenen Lebererkrankung [5]. Mithilfe einer hochwertigen MRCP sind auch Gallengänge dritter Ordnung darstellbar. Eine additive MR-Elastographie ermöglicht die Graduierung der Leberfibrose und Prognoseeinschätzung. Zu den Einschränkungen der MRCP zählt die verminderte Darstellbarkeit intrahepatischer kleiner Gallengänge, was insbesondere die Möglichkeit einer frühen Diagnose limitiert. Auch falsch-positive Befunde durch unspezifische Gallengangsveränderungen bei Lebererkrankungen jeglicher Ätiologie sind möglich.

**Figure 2:**
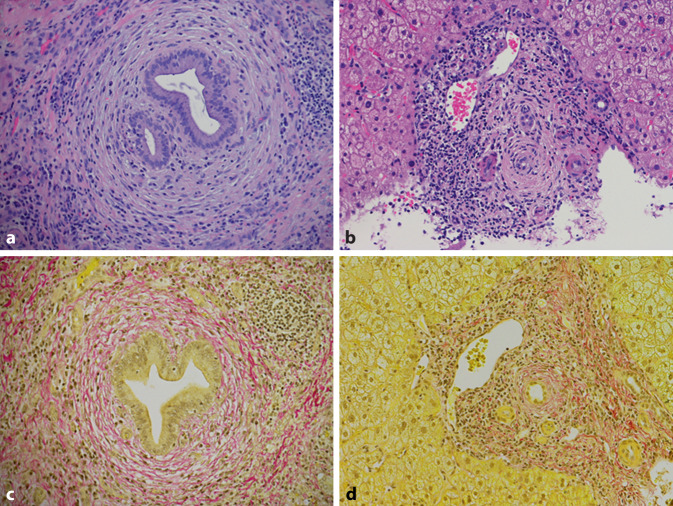

Eine Leberbiopsie ist für die Diagnose bei PatientInnen mit PSC-typischen cholangiographischen Veränderungen nicht erforderlich. In etwa 10 % der Fälle betrifft die PSC im Sinne einer Small-duct-PSC nur die kleinen Gallenwege, die mit den derzeitigen MRT- und ERCP-Techniken nicht darstellbar sind. In diesen Fällen ist eine Leberbiopsie für die Diagnosestellung erforderlich (Abb. 2). Bis zu 23 % der Small-duct-PSC gehen innerhalb von 8 Jahren in eine Large-duct-PSC über [4]. Die typischen histologischen Veränderungen (z. B. periduktale Zwiebelschalenfibrose) sind nicht pathognomonisch für die PSC und werden beispielsweise auch bei mit Mutationen der Phospholipidexportpumpe ABCB4 assoziierten Erkrankungen beobachtet. Die Verdachtsdiagnose einer Small-duct-PSC ist daher stets kritisch zu hinterfragen, insbesondere dann, wenn keine CED vorliegt. Neben der Diagnostik einer Small-duct-PSC kann die Leberbiopsie zum Ausschluss eines PSC-Autoimmunhepatitis(AIH)-Überlappungssyndroms notwendig sein. Ein solches sollte bei PSC-PatientInnen mit Alanin-Aminotransferase(ALT)-Werten über dem 5‑fachen oberen Normwert (ULN) und/oder IgG-Spiegel über dem 1,5-fachen des oberen Normwerts suspiziert werden [4].

Die Diagnose einer PSC kann erst gestellt werden, nachdem mögliche Ursachen einer sekundär sklerosierenden Cholangitis ausgeschlossen wurden (siehe Tab. 1). Hier ist insbesondere die IgG4-assoziierte Cholangitis (IAC), die mit Gallengangsstrikturen, erhöhten Cholestasefermenten und einer Erhöhung des Serum-IgG4 einhergeht, hervorzuheben. Da in bis zu 15 % der PSC-PatientInnen IgG4-Erhöhungen beobachtet werden, kann die Unterscheidung zwischen IAC und PSC schwierig sein [6]. Charakteristischerweise wird die IAC bei älteren Männern gefunden, die über einen längeren Zeitraum potenziell schädlichen Chemikalien, insbesondere organischen Lösungsmitteln, ausgesetzt waren [4]. Die Diagnose einer IAC kann mithilfe der HISORt-Kriterien gestellt werden, die auf histologischen (H), bildgebenden (I) und serologischen (IgG4) Befunden (S), einer Beteiligung anderer Organe (unter anderem Autoimmunpankreatitis, Sialadenitis; O) und dem Ansprechen auf eine Glukokortikoidtherapie (Rt) basieren [4]. Im Vergleich zu den charakteristischen „Skip“-Strikturen bei der PSC sind die Gallengangsalterationen bei IAC häufig länger und bandförmig. Neben der PSC stellt auch das CCC eine relevante Differenzialdiagnose der IAC dar. Die Kriterien, die eine Unterscheidung zwischen IAC und CCC erleichtern können, sind in Tab. 2 zusammengefasst.

**Table 1:** 

	Erkrankung
Chronisch obstruktiv	Choledocholithiasis
	Cholangiozelluläres Karzinom
	Lebermetastasen
	Portal hypertensive Biliopathie
	Gallengangsstrikturen nach Operationen (z. B. nach LTx, CHE)
	Chronische Pankreatitis
Ischämisch/vaskulär	Verletzung oder Thrombose der A. hepatica nach CHE
	Nichtanastomotische Stenosen nach LTx (z. B. Allograft-Abstoßung)
	(Intraarterielle) Chemoembolisation
	Kleingefäßvaskulitis
	Systemische Hypoxämie
	Low-output-Syndrom
	SIRS/Sepsis
	Polytrauma/Verbrennung
Immunologisch	IgG4-assoziierte Cholangitis
	Eosinophile Cholangitis
	Mastzellencholangiopathie
	Systemischer Lupus erythematodes
	Sarkoidose
	Allograft-Abstoßung nach LTx
Infektiös	Kryptosporidien
	Mikrosporidien
	Zytomegalievirus
	AIDS-assoziierte Cholangiopathie
	*Clonorchis sinensis*
	Rezidivierende bakterielle Cholangitiden
Medikamentös-toxisch	Ethanol (akzidentelle biliäre Applikation)
	Immuncheckpointinhibitor(ICI)-induzierte Cholangitis
	Ketamin
Hereditär	Zystische-Fibrose-assoziierte Cholangiopathie
	Genetisch bedingter ABCB4-Mangel
Neoplastisch	M. Hodgkin
	Histiozytose X

*ABCB4* hepatokanalikulärer Phospholipidtransporter, *CHE* Cholezystektomie, *LTx* Lebertransplantation, *SIRS* systemisch-inflammatorisches Antwortsyndrom

**Table 2:** 

Methode	PSC	IAC	CCC
*Klinische Präsentation*	– Männer:Frauen 1,3:1 – Alter < 40 – Oligosymptomatisch, cholestatische Leberenzymerhöhung oft ohne Ikterus – Cholangitis	– Männer:Frauen 1,7:1 – Alter > 50 – Ikterus – Gewichtsverlust	– Männer:Frauen 1,3:1 – Alter 60–80 – Ikterus – Gewichtsverlust – Dumpfe Schmerzen
*Labor*	– Erhöhung von Serum-IgG4 in 15 % PSC-PatientInnen – Erhöhung von CA19‑9 in 30 % der PSC mit dominanter Stenose ohne Neoplasie	*Serum-IgG4* – > 4 × ULN (100 % Spezifität) – 1–2 × ULN: IgG4/IgG1-Verhältnis > 0,24, weist auf IAC hin *Serum-IgG2 hoch*	CA19‑9 kein guter diagnostischer Marker
*Bildgebung:* *MRCP* *Endosonographie* *PET-CT*	– Umschriebene kurze Strikturen – Perlenförmiger Gallenbaum – Gestutzter („pruned“) Gallengangsbaum – Übersprungene Gallengangsläsionen – Divertikel – Wanddicke des DHC < 2,5 mm	– Lange, bandförmige Strikturen – Fehlen von kurzen Gallengangsstrikturen – Wanddicke von DHC > 2,5 mm – Kontinuierliche Wanddicke des Gallengangs vom distalen Gallengang bis zur Hilusregion	– Kurze Gallengangsstriktur – Raumforderung
*Andere Besonderheiten*	CED	Beteiligung von Pankreas und anderen Organen (Speicheldrüse, Schilddrüse, Niere)	Metastasen

*CA19‑9* „carbohydrate antigen 19-9“, *CCC* cholangiozelluläres Karzinom, *CED* chronisch-entzündliche Darmerkrankung, *DHC* Ductus hepatocholedochus, *IAC* IgG4-assoziierte Cholangitis, *PSC* primär sklerosierende Cholangitis

Bei PSC-PatientInnen werden häufig Autoantikörper nachgewiesen; antinukleäre Antikörper (ANA) bei 8–77 %, Antikörper gegen glatte Muskeln (ASMA) bei bis zu 83 % und antineutrophile zytoplasmatische Antikörper (ANCA) bei 26–96 % [4]. Letztere werden bei PSC nachgewiesen, ohne dass diesen Antikörpern eine diagnostische Spezifität zukommt. Diese haben aber möglicherweise prognostische Relevanz [4]. Die Bestimmung von ANA und ASMA wird bei Verdacht auf PSC-AIH-Overlap empfohlen [4].

Bei PSC-Verdacht kann die Diagnostik einer CED richtungsweisend sein. Daher soll auch bei PatientInnen ohne bereits bekannte CED eine Koloskopie mit Stufenbiopsien durchgeführt werden, da die CED bei PSC meist einen spezifischen Phänotyp aufweist, der durch einen milden, gelegentlich auch gänzlich asymptomatischen Krankheitsverlauf, eine vorrangig rechtsseitig betonte Entzündung, Rektumaussparung und ein hohes CRC-Risiko charakterisiert ist. Endoskopisch kann die Schleimhaut mitunter normal erscheinen, während die Histologie eine zugrunde liegende milde CED offenbart. Das erhöhte CRC-Risiko und die oft oligosymptomatische Erkrankung machen eine Koloskopie mit Biopsien bei allen PatientInnen bei PSC-Diagnose unerlässlich. Bei initial unauffälliger Koloskopie ist eine Wiederholung innerhalb von 5 Jahren angezeigt [7].

## Prognoseeinschätzung, Risikostratifizierung und Verlaufskontrolle

Der Krankheitsverlauf der PSC ist interindividuell variabel und reicht von milden, relativ asymptomatischen Verläufen ohne Komplikationen bis ins hohe PatientInnenalter bis hin zu schweren rezidivierenden Cholangitiden, biliärer Zirrhose, CCC oder der Notwendigkeit zur Lebertransplantation (LTx) in bereits jungen Jahren. Die Mortalität ist bei PSC im Vergleich zur Normalbevölkerung etwa um das 4‑fache erhöht [8]. Ein junges Alter bei Diagnosestellung, weibliches Geschlecht, das Vorliegen einer Small-duct-PSC, ein Morbus Crohn und eine normale oder nur gering erhöhte AP (mit oder ohne Ursodesoxycholsäure[UDCA]-Therapie) gehen mit einer guten Prognose einher [1]. Ein spontaner oder durch UDCA bedingter Rückgang der AP war in Studien mit einem signifikanten Überlebensvorteil assoziiert, kann jedoch angesichts unzureichender Evidenz nicht als alleiniger Surrogatparameter empfohlen werden [4]. Das Auftreten eines Ikterus, eines niedrigen Serumalbumins, eine verminderte Thrombozytenzahl und/oder eingeschränkte Blutgerinnung sind wie bei anderen Lebererkrankungen prognostisch als schlecht zu werten. Diese treten bei PSC allerdings erst in fortgeschrittenen Stadien auf. Eine exakte Prognoseeinschätzung stellt uns daher vor eine unzureichend gelöste Herausforderung, vor allem in Bezug auf den optimalen Zeitpunkt der LTx und die individuelle Risikoeinschätzung für die malignen Komplikationen CCC und CRC, die unabhängig von der Schwere der Lebererkrankung auftreten.

Nichtinvasive Fibrosetests (transiente Elastographie, ELF[„enhanced liver fibrosis“]-Test, Fib4) stellen attraktive, nichtinvasive Möglichkeiten zur verbesserten Prognoseeinschätzung bei PSC dar [4]. Ein ELF-Test mit Werten < 7,7 war mit einer äußert niedrigen Rate an leberspezifischen Komplikationen und einer exzellenten Prognose verbunden [9]. Mittels Elastographie konnte gezeigt werden, dass ein jährlicher Anstieg um > 1,5 kPa bzw. ein Ausgangswert von > 9,9 kPa mit einer schlechteren Prognose assoziiert war [10]. Hingegen war ein Ausgangswert von < 6,5 kPa mit einer niedrigen Komplikationsrate verbunden [10]. Anzumerken ist, dass eine mechanische Cholestase insbesondere im rechten Leberlappen falsch hohe Elastizitätswerte erzielen und damit zu einer falschen Prognoseeinschätzung führen kann [11].

Auf MRT basierende Scores wie der ANALI-Score [12, 13] und DiStrict-Score [14] eignen sich dazu, prognostisch relevante Veränderungen des Leberparenchyms und der Gallengänge objektiv zu erfassen, werden aber selten verwendet. Der Tauglichkeitsnachweis dieser Scores für die Prognoseeinschätzung in der klinischen Routine wird zu erbringen sein. Relativ rezent wurde die Tauglichkeit der MRC+-Software als automatisches Post-processing-Softwaretool zur objektiven Quantifizierung von mittels MRCP detektierten Strikturen und Dilatationen in Hinblick auf die Prognoseeinschätzung bei PSC untersucht [15, 16]. MRC+ ist aktuell primär für Studienzwecke attraktiv. Simple Parameter wie die Milzgröße/-länge sind ebenfalls von prognostischer Bedeutung [4].

Die European Association for the Study of the Liver (EASL PSC Clinical Practice Guidelines 2022) empfiehlt bereits bei Diagnosestellung eine Risikostratifizierung basierend auf nichtinvasiven Fibrosetests (NIT) und Daten zum Erkrankungsphänotyp (AP, Bilirubin, Albumin, Thrombozyten und Gerinnungstests, MR/MRCP-Befunde; [4]). Für die PSC wurden unterschiedliche Prognosemodelle wie der PSC-spezifische Mayo PSC Risk Score oder das „Primary Sclerosing Cholangitis Risk Estimate Tool“ (PREsTo) entwickelt [17]. Auch Webtools wie Amsterdam-Oxford PSC Score mit Option des „online calculator“ (Link: https://sorted.co/psc-calculator/) können hilfreich sein [18]. Der folgende vorgeschlagene auf den aktuellen EASL Guidelines beruhende simple Algorithmus zur Risikostratifizierung erscheint im Vergleich zu den oben genannten Scores einfacher und klinisch gut praktikabel (Tab. 3).

**Table 3:** 

**Niedriges Risiko**
Small-duct-PSC ohne Zirrhose **ODER** Klassische PSC mit folgenden phänotypischen Charakteristika: Asymptomatisch *UND* Bilirubin, Albumin, Thrombozyten und Gerinnungstests normal *UND* AP < 1,5 × ULN *UND* LSM (transiente Elastographie) < 6,5 kPa oder ELF-Test < 7,7 *UND* Wenig ausgeprägter Befund an den Gallenwegen in der MR/MRCP
**Signifikantes Risiko**
Symptomatisch *ODER* Bilirubin, Albumin, Thrombozyten oder Gerinnungstests pathologisch *ODER* AP > 1,5 × ULN *ODER* LSM (transiente Elastographie) > 9,9 kPa oder ELF-Test > 10,6 *ODER* Ausgeprägter Befund an den Gallenwegen in der MR/MRCP

*AP* alkalische Phosphatase, *ELF-Test* Enhanced-liver-fibrosis-Test, *kPa* Kilopascal, *LSM* Lebersteifigkeitsmessung, *MR/MRCP* Magnetresonanz/Magnetresonanzcholangiopankreatikographie, *PSC* primär sklerosierende Cholangitis, *ULN* obere Normgrenze

**Table 4:** 

*Empfohlene Routineuntersuchungen/-kontrollen bei PSC*
Klinische Untersuchung und Labor (Bilirubin, AP, GGT, AST, ALT, TZ, INR) alle 6–12 Monate (in Abhängigkeit von der Risikoeinstufung)
MR/MRCP und/oder Ultraschall (mit speziellem Fokus auf die Gallenblase) alle 12 Monate (Ultraschall alle 6 Monate bei Zirrhose zum HCC Sceening)
Koloskopie alle 12 Monate bei CED, alle 5 Jahre wenn keine CED
LSM mittels Elastographie und/oder ELF-Test alle 12 Monate
Knochendichtemessung alle 2–4 Jahre
*Bei klinischer Verschlechterung, Laborveränderungen (Anstieg Bilirubin u./o. AP) oder ∆LSM >* *1,5* *kPa/Jahr*
Serum-CA19‑9 und MR/MRCP mit Kontrastmittel und ERCP inkl. Zytologie/Histologie/FISH bei V. a. CCC
Bei ALT/AST-Anstieg bzw. V. a. AIH-Overlap Serum-IgG und Autoantikörper und ggf. Leberbiopsie
Bei Zeichen der portalen Hypertension Vorgehen laut Baveno-VII-Konsensus

*AIH* Autoimmunhepatitis, *ALT* Alanin-Aminotransferase, *AP* alkalische Phosphatase, *AST* Aspartat-Aminotransferase, *ELF-Test* Enhanced-liver-fibrosis-Test, *GGT* γ-Glutamyltransferase, *kPa* Kilopascal, *LSM* Lebersteifigkeitsmessung, *MR/MRCP* Magnetresonanz/Magnetresonanzcholangiopankreatikographie, *PSC* primär sklerosierende Cholangitis, *TZ* Thrombozyten

Diese einfache Risikostratifizierung sollte im Rahmen der Verlaufskontrollen erfolgen. PatientInnen mit signifikantem Risiko sollten engmaschig in 3‑ bis 6‑monatlichen Intervallen kontrolliert werden, während bei niedrigem Risiko und klinisch stabilem Befund eine klinische Kontrolle (Fragen zu Lebensqualität wie Pruritus, Fatigue) sowie Überprüfung der Laborparameter (AP, GGT, AST, ALT, Bilirubin, Albumin, Thrombozyten und Gerinnungstests) alle 12 Monate ausreicht (Tab. 3 und 4). Die transiente Elastographie oder der ELF-Test sollte jährlich erfolgen. Bei einem jährlichen Anstieg der Lebersteifigkeit um 1,5 kPa sollten die Kontrollintervalle (Klinik/Labor/Bildgebung) verkürzt werden, um eine Dekompensation frühzeitig zu erkennen [4].

Die aktuell gültigen Leitlinien von EASL und Deutscher Gesellschaft für Gastroenterologie, Verdauungs- und Stoffwechselkrankheiten (DGVS) empfehlen zudem alle 12 Monate eine Bildgebung (Abdomensonographie und/oder MR/MRCP) mit besonderer Berücksichtigung der Gallenblasenwand [4, 19]. Sollte es hier zum Auftreten von Gallenblasenpolypen kommen, sollten diese regelmäßig sonographisch überwacht und bei einer Größe über 8 mm oder Größenprogredienz die Cholezystektomieindikationsstellung nach sorgfältiger Nutzen-Risiko-Abwägung in Bezug auf die individuelle Leberfunktion und das Ausmaß des portalen Hypertonus erfolgen. Hier ist anzumerken, dass relativ rezente sowohl human als auch im Tiermodell generierte Daten auf eine mögliche protektive Rolle der Gallenblase bei PSC hinweisen [20].

Bei Vorliegen einer Leberzirrhose sollte halbjährlich eine Sonographie zur Surveillance eines hepatozellulären Karzinoms (HCC) erfolgen. Was die frühzeitige Detektion eines CCC mittels jährlicher Bildgebung betrifft, so ist die Datenlage unklar. In einer 2023 publizierten prospektiven Beobachtungsstudie aus Schweden konnte kein Vorteil für die jährliche Durchführung einer MRT mit gleichzeitiger Bestimmung von CA19‑9 zur Prognoseverbesserung bei CCC gezeigt werden [21]. Eine wirksame CCC-Surveillance-Strategie bei PSC bleibt daher aktuell Wunschdenken. Surveillance-Koloskopien sollen bei PatientInnen mit gleichzeitiger CED jährlich ab dem Zeitpunkt der PSC-Diagnosestellung durchgeführt werden. [19]. Die erschreckenden Ergebnisse einer Umfrage zur Durchführung von Surveillance-Koloskopien bei PSC müssen uns wachrütteln, denn 27 % der Befragten empfahlen diese nur im Falle von Symptomen [22]. Häufig besteht eine Einschränkung der Lebensqualität durch chronische Müdigkeit oder Pruritus. Zudem liegt die Prävalenz für Osteopenie bei 40 % und die der Osteoporose bei 11–15 % [4]. Eine Knochendichtemessung wird daher im Abstand von 2 bis 4 Jahren empfohlen [19].

## Medikamentöse Therapieoptionen

Derzeit existiert noch keine etablierte medikamentöse Therapie der PSC. In vielen Ländern, so auch in Deutschland, Österreich und der Schweiz, kommt analog zu anderen cholestatischen Erkrankungen bei der PSC die Ursodesoxycholsäure (UDCA) als hydrophile (damit weniger toxische) Gallensäure mit choleretischen, zytoprotektiven und antiinflammatorischen Eigenschaften zum Einsatz [23], obwohl dadurch noch kein Effekt auf das transplantationsfreie Überleben bei PSC gezeigt werden konnte [5]. In einzelnen Ländern wie Frankreich und der Schweiz ist UDCA zur Therapie der PSC zugelassen. In anderen Ländern erfolgt die Gabe bei PSC somit „off label“ [5]. Entsprechend den aktuellen Empfehlungen der Fachgesellschaften (AASLD, EASL, DGVS) kann UDCA bei PSC in einer mittleren Dosierung von 15 bis 20 mg/kg gegeben werden [4, 19, 24]. Eine hoch dosierte (28–30 mg/kg/d) Therapie sollte aufgrund der negativen Auswirkungen auf hepatologische Endpunkte und das kolorektale Dysplasierisiko nicht zum Einsatz kommen. Obwohl das biochemische Ansprechen bei PSC mit keinem klaren Überlebensvorteil assoziiert ist, stellt die Orientierung an der biochemischen „response“ (z. B. AP-Reduktion um 40 %) als Entscheidungskriterium für das Fortsetzen einer UDCA-Therapie eine klinisch durchaus plausible/pragmatische Vorgangsweise dar [24, 25]. Ein positiver Effekt von UDCA auf das transplantationsfreie Überleben konnte weder in zwei Metaanalysen noch in einer skandinavischen (der bisher größten randomisierten, placebokontrollierten) Studie an 219 PSC-PatientInnen nachgewiesen werden [5]. Eine rezente japanische Registerstudie mit 435 PatientInnen zeigt eine reduzierte Mortalität bzw. Transplantationsrate unter UDCA [26]. Die Aussagekraft dieser Studie wird angesichts ihres retrospektiven Charakters und eines möglichen Selektionsbias und weiterer Variablen wie der gleichzeitigen Gabe von Fibraten kontroversiell diskutiert.

Die Daten zur möglichen Prävention von CRC und CCC durch UDCA sind zu uneindeutig, um davon eine klare Therapieempfehlung abzuleiten [5]. In zwei Metaanalysen konnte keine signifikante CRC-Reduktion beobachtet werden, wobei unter Berücksichtigung von fortgeschrittenen Läsionen (CRC und hochgradige Dysplasien) in einer der Metaanalysen ein positiver Effekt beobachtet wurde. Auch hier gilt die Vermeidung höherer UDCA-Dosierungen (28–30 mg/kg), da hierbei sogar eine erhöhte Rate kolorektaler Dysplasien auftrat [4]. Die derzeit laufenden Studien testen eine Vielzahl von Therapieansätzen wie Norucholsäure (vormals norUDCA), Inhibitoren des Gallensäuretransportsystems im Ileum (IBAT-Inhibitoren), Fibrate (und andere spezifischere PPAR-Liganden), Statine, Vancomycin und Bexotegrast (Integrininhibitor). Eine rezente Phase-3-Studie mit Cilofexor (FXR-Agonist) war leider negativ.

Der Einsatz von Glukokortikoiden, Immunsuppressiva und Biologika wird für die Routinebehandlung der PSC nicht empfohlen [5]. Ausnahmen stellen hier PatientInnen mit PSC-AIH-Overlap-Syndrom bzw. PatientInnen mit einer IAC dar. Glukokortikoide oder Immunsuppressiva werden bei PSC-PatientInnen mit geringgradig erhöhtem Serum-IgG4 (< 2 × ULN) ohne klares Vorliegen aller Kriterien für eine IAC dezidiert nicht empfohlen [5]. Der Einsatz von Biologika zur Therapie der CED bei PSC hat keinen klaren Effekt von Anti-TNF-Antikörpern wie Infliximab oder Integrinblockern wie Vedolizumab auf serumbiochemische Auslenkungen bei PSC gezeigt, möglicherweise besteht hier ein schwaches positives Signal für Adalimumab. Allerdings wurde über ein erhöhtes bakterielles Cholangitisrisiko unter Anti-TNF-Antikörpern (nicht aber unter Immunmodulatoren/Azathioprin) bei PSC berichtet [27]. Neuere immunologische/antiinflammatorische Therapieansätze wie JAK-Inhibitoren und Anti-IL-23-Strategien haben in Pilotstudien für Tofacitinib [28] erste positive Signale gezeigt und werden derzeit im Rahmen klinischer Studien noch weiter untersucht.

Die Langzeitgabe von Antibiotika (inkl. Vancomycin) wird für die Behandlung der PSC nicht empfohlen [5]. Die Antibiotikatherapie bleibt daher auf die Therapie akuter bzw. rekurrierender bakterieller Cholangitiden und die Cholangitisprophylaxe bei der ERCP beschränkt. Die Daten zu Vancomycin bei CED und PSC sind vielversprechend, wobei eine rezente Studie bei Kindern mit PSC keinen „benefit“ gezeigt hat [29]. Entsprechende prospektive, kontrollierte Studien bei Erwachsenen sind noch ausständig. Andere Antibiotika (z. B. Metronidazol) haben in Kombination mit UDCA zwar positive Signale gezeigt, können aber aufgrund der Datenlage aktuell nicht empfohlen werden. Daten zur Stuhltransplantation sind auf eine kleine Pilotstudie beschränkt und sind ebenfalls als experimentell einzustufen. Akute bakterielle Cholangitiden sollten mit Antibiotika und bei Vorliegen relevanter Strikturen als deren Ursache endoskopisch therapiert werden. Ein Problem stellen rekurrierende Cholangitiden dar, welche insbesondere in der Überbrückung bis zur LTx oft einer Therapie mit rotierenden Antibiotikazyklen bedürfen. Fluorchinolone sollten dabei aufgrund der veränderten Resistenzlage und ihres ungünstigen Nebenwirkungsprofils nur mehr in Ausnahmefällen zum Einsatz kommen. Unter Berücksichtigung des lokalen Keimspektrums und dessen Resistenzlage ist primär der Einsatz von Aminopenicillinen/β-Laktamase-Hemmern bzw. Piperacillin/Tazobactam oder in weiterer Folge Cephalosporinen der dritten Generation angezeigt.

In der Therapie des Pruritus bei PSC sollen entsprechend den neuen EASL Guidelines primär Fibrate (Bezafibrat 4 mg/d) zum Einsatz kommen, in weiterer Folge Rifampicin (150–300 mg/d) und/oder Naltrexon (12,5–50 mg/d; [5]). Aufgrund der fehlenden Evidenz werden Anionenaustauschharze zur medikamentösen Therapie des Pruritus bei PSC nicht mehr empfohlen. Für Fibrate spricht neben dem antipruritogenen Effekt aufgrund erster Pilotdaten auch ein möglicher positiver Effekt auf den Krankheitsverlauf analog zu den Daten bei PBC; entsprechende Studien sind noch nicht abgeschlossen.

## Endoskopische Therapie

Die endoskopische Therapie der PSC zielt auf Symptomlinderung und Verbesserung der mechanischen Cholestase, z. B. bei Vorliegen einer bakteriellen Cholangitis infolge von Strikturen, ab. Im klinischen Alltag stellen sowohl die Diagnostik solcher Strikturen als auch die Unterscheidung von benignen und malignen Strikturen eine der größten Herausforderungen dar. Die Schwierigkeit mit dem neuen klinischen Terminus der *relevanten* (früher „dominanten“) *Striktur* beginnt bei dessen Definition. Während *dominante Strikturen* für viele Jahre als Stenosen mit einem (mittels ERCP gemessenen) Durchmesser von ≤ 1,5 mm im Bereich des Ductus hepatocholedochus (DHC) bzw. ≤ 1 mm in einem der beiden Hepatikushauptäste definiert waren [7], spricht man aktuell von einer *relevanten Striktur* bei einer hochgradigen Striktur (einer > 75 %igen Reduktion des Gangdurchmessers entsprechend) des DHC oder eines der beiden Hepatikusäste in der Bildgebung (MRCP) bei auftretenden Symptomen einer obstruktiven Cholestase (Fieber, Juckreiz, Ikterus) und/oder bakteriellen Cholangitis [4, 5]. Die Entwicklung erfolgte weg von einer rein durch Bildgebung gestellten Diagnose hin zu einer klinisch *und* radiologisch (i.e. funktionell) definierten. Ob sich dies bewährt, wird noch klar zu zeigen sein.

Die endoskopisch-interventionelle Therapie relevanter Stenosen beinhaltet Verfahren wie die hydrostatische Dilatation (auf 6–8 mm), die Bougierung, die (kurzzeitige) Einlage von Plastik- oder beschichteten Metallstents oder die Kombinationen dieser Verfahren [19]. Ein standardisiertes Vorgehen basierend auf einer deutlichen Überlegenheit eines Verfahrens in hochqualitativen randomisierten Studien steht uns bislang nicht zur Verfügung. Studiendaten zu Kurzzeitstenting und Ballondilatation zur Therapie dominanter Strikturen belegen eine vergleichbare Effektivität beider Verfahren, wenngleich die Stenteinlage häufiger mit dem Auftreten bakterieller Cholangitiden assoziiert zu sein scheint [30, 31]. Daraus resultierend empfehlen die aktuell gültigen EASL-CPG eine wiederholte Dilatation (2- bis 3‑mal in 1‑ bis 4‑wöchigen Abständen) und möglichst kurzzeitige Stenteinlage mit einer maximal 2‑ bis 4‑wöchigen Liegedauer – sofern die Komplexität der Striktur eine solche überhaupt erfordert [4]. Neben den häufig verwendeten Plastikstents wurden rezent auch vollbeschichtete, wiederentfernbare Metallstents (Verweilzeit 3 Monate) in einer retrospektiven Fallserie an 20 PatientInnen untersucht, wobei sich durchaus gute Ergebnisse zeigten [32]. Zur perkutanen Drainage, die in komplexen Fällen erforderlich sein kann, liegen bei PSC sehr begrenzte Daten vor.

Wenn endoskopische Interventionen bei PSC erfolgen, sollte gemäß aktuellen Empfehlungen der Fachgesellschaften eine periinterventionelle Antibiotikaprophylaxe durchgeführt werden. Dies gilt insbesondere dann, wenn eine komplexe Gallengangsschädigung (z. B. durch Dilatation) oder eine unvollständige Drainage zu erwarten ist [4, 7, 19]. Die ERCP stellt – unabhängig davon, bei welcher Indikation sie durchgeführt wird – einen wesentlichen Risikofaktor für das Einbringen von Keimen in das Gallengangssystem dar. Auch scheint gerade bei PSC die Cholangitisrate im Vergleich zu anderen Erkrankungen selbst nach der Gabe einer prophylaktischen antibiotischen Therapie erhöht zu sein [33]. Ein Zusammenhang bakterieller Cholangitiden mit der Krankheitsprogression wird immer wieder vermutet, bleibt aktuell aber spekulativ [4]. Ob eine periinterventionelle Antibiose einen „benefit“ in Hinblick auf die Mortalität bringt, wie lange und mit welchen antimikrobiellen Substanzen sie durchgeführt werden sollte und wann diese gestartet werden sollten (bereits vor oder erst nach der Intervention), kann durch die aktuelle Studienlage dazu nicht hinreichend klar beantwortet werden [7, 19]. Die Wahl des Antibiotikums sollte das lokale und patientInnenspezifische Resistenzspektrum miteinbeziehen. Eine mikrobiologische Untersuchung der Galle erscheint in der Auswahl bzw. nachfolgenden Adaptation des Antibiotikaregimes vorteilhaft und wird in diesem Setting auch empfohlen [19]. Ob sich eine wiederholte, prophylaktische, periinterventionelle Antibiose bei PSC auch nachteilig im Sinne der Selektion resistenter Keime auswirken kann, ist bis dato unzureichend untersucht.

Die European Society for Gastrointestinal Endoscopy (ESGE) empfiehlt zur Vermeidung einer Post-ERCP-Pankreatitis die prophylaktische rektale Verabreichung von Diclofenac oder Indometacin auch für PSC-PatientInnen [7]. Ob die Diclofenacgabe für PSC-PatientInnen bzgl. der Post-ERCP-Pankreatitis-Rate bei PSC-PatientInnen von etwa 5 bis 7 % [34, 35] tatsächlich einen Vorteil bringt, wurde rezent in einem finnischen PSC-Kollektiv retrospektiv untersucht. In dieser Studie wurde kein signifikanter Unterschied im Auftreten bzw. der Schwere der Post-ERCP-Pankreatitis mit und ohne Diclofenacgabe gefunden. Die Autoren wiesen jedoch darauf hin, dass ohne ausreichend dimensionierte RCT keine Änderung der Empfehlungen ausgesprochen werden kann [35]. Eine Sphinkterotomie bei ERC sollte aufgrund des damit verbundenen erhöhten Risikos einer aszendierenden Cholangitis bei PSC möglichst vermieden und, wenn erforderlich, so klein als möglich durchgeführt werden [7].

Die Indikation zur diagnostischen ERC beschränkt sich bei PSC auf die Abklärung malignitätsverdächtiger Strikturen. Etwa 10–20 % aller PSC-PatientInnen entwickeln ein CCC. Malignitätsverdächtig sind insbesondere relevante Strikturen bei neu bzw. rezent diagnostizierter PSC (30–35 % aller CCC werden im ersten Jahr nach Erstdiagnose der PSC diagnostiziert [8]) bzw. eine Verschlechterung infolge einer Striktur oder neu aufgetretene Raumforderungen in der Leber oder im Bereich der extrahepatischen Gallenwege bei bestehender PSC [4]. Die CCC bei PSC sind meist perihilär lokalisiert, kommen aber auch weiter distal (nach Einmündung des D. cysticus) und intrahepatisch (jenseits der Gallengänge zweiter Ordnung) vor. Das Management in der Abklärung dieser potenziell malignitätsverdächtigen Stenosen beinhaltet die endoskopische Probenentnahme (zytologische Abstriche und Biopsie). Die Bürstenzytologie ist die am besten evaluierte Methode, weist allerdings eine niedrige Sensitivität auf [36]. Bei nicht eindeutigen Ergebnissen in der Bürstenzytologie kann die Detektion von DNA-Aberrationen mittels Fluoreszenz-in-situ-Hybridisierung (FISH) als zusätzliche Methodik herangezogen werden [37]. Diese Methode steht nur in wenigen Zentren standardisiert als Routineverfahren zur Verfügung. Weitere sehr elegante Methoden sind die Cholangioskopie mit gezielter Biopsie (diagnostische Treffsicherheit 96 % [38]), die konfokale Lasermikroskopie und der endoskopische Ultraschall inkl. Feinnadelaspiration [4]. Die klinische Wertigkeit dieser Methoden kann noch nicht ausreichend beurteilt werden, die Methoden scheinen aber vielversprechend.

## Lebertransplantation

Die Lebertransplantation (LTx) stellt die einzige kurative Therapie für PSC-PatientInnen mit fortgeschrittener Erkrankung dar. Eine rezente Analyse aus dem europäischen LTx-Register (ELTR) von 1549 transplantierten PSC-PatientInnen (1980–2015) zeigte ein Organüberleben nach 1, 5, 10 und 20 Jahren von hervorragenden 80, 70, 60 und 41 %. Eine LTx-Evaluierung sollte nach zumindest 2 spontanen Episoden einer septischen Cholangitis innerhalb von 6 Monaten, bei Dekompensation der Lebererkrankung, dem Auftreten eines HCC (entsprechend den HCC-Standardkriterien) und bei Auftreten einer relevanten Gallengangsstriktur mit endoskopisch/interventionell unzureichender Behandlungsmöglichkeit bzw. persistierendem Juckreiz und obstruktiver Cholestase mit persistierend > 6 mg/dl erhöhtem Serumbilirubin über 6 Monate erfolgen [4]. Eine wichtige und weitgehend ungelöste Frage ist die Indikationsstellung zur LTx bei Vorliegen einer endoskopisch/zytologisch/histologisch nachgewiesenen hochgradigen Dysplasie des Gallengangsepithels. In Fallstudien zu dieser Frage wurden in 20–57 % der Fälle keine CCC in den explantierten Organen gefunden, was als rechtzeitige Lebertransplantation bei drohendem CCC, aber in manchen Fällen auch als Überbehandlung mit Lebertransplantation interpretiert werden kann. Im Falle des Vorliegens eines CCC bei PSC ist die interdisziplinäre Falldiskussion in einem spezialisierten Zentrum zur bestmöglichen Therapiestrategieplanung unumgänglich. Sollten solche PatientInnen KandidatInnen für eine LTx sein, empfiehlt die aktuelle EASL-CPG, dies ausschließlich im Rahmen von klinischen Studien durchzuführen [4]. Entscheidend für gute Gesamtergebnisse bei dieser Indikation ist dabei vor allem eine sehr sorgfältige PatientInnenselektion.

Bei der PSC stellt das Problem eines Wiederauftretens der Erkrankung nach Transplantation mit einer Rate von 17 % in der oben erwähnten ELTR-Analyse – in anderen Analysen bis zu 25 % – ein im Vergleich zu anderen cholestatischen Lebererkrankungen wie der PBC deutlich prominenteres Problem dar. Die Diagnose eines PSC-Rezidivs im Transplantat sollte anhand der Graziadei-Kriterien erfolgen [4]. Diese schließen neben anderen wichtigen Faktoren vor allem auch den möglichst sicheren Ausschluss eines arteriellen Versorgungsproblems des Transplantats ein. In vielen Fällen einer wiederauftretenden PSC wird das Problem nur mittels einer Retransplantation, die vor allem bei einem kalkulierten 5‑Jahres-Überleben von > 50 % verfolgt werden sollte, behoben werden können. Insgesamt gilt die LTx als Standardtherapieverfahren der fortgeschrittenen PSC.

## Fazit für die Praxis

Die Diagnostik und Prognosebeurteilung bei PSC wurden durch MRT-basierte Methoden und die erfolgreiche Einführung von NIT deutlich verbessert. Die Suche nach geeigneten Biomarkern bei PSC war bislang wenig erfolgreich. Mithilfe von internationalen Studiengruppen, großen Datensätzen und modernen selbstlernenden Programmen zur Erstellung solcher Modelle sind weitere Verbesserungen zu erwarten. Der Einsatz und die Zielsetzung der Endoskopie bei PSC haben sich deutlich gewandelt und zu einer weiteren Risikominimierung unserer PatientInnen beigetragen, die Indikation zur ERCP beschränkt sich auf die Abklärung malignitätsverdächtiger und Therapie (z. B. Ballondilatation, Stenteinlage) relevanter Strikturen. Die Evidenz für Empfehlungen zur medikamentösen Therapie (z. B. Ursodesoxycholsäure) bei PSC bleibt gering. Ergebnisse von mehreren Phase-II-Studien zur medikamentösen Therapie sind vielversprechend. Die Ergebnisse von mehreren Phase-III-Studien werden 2024/2025 mit Spannung erwartet. Die LTx liefert bei PSC gute Langzeitergebnisse, ist aber mit einem beträchtlichen Rezidivrisiko behaftet. Aufgrund des stark erhöhten Risikos für CRC werden jährliche Koloskopien für PSC-PatientInnen mit CED empfohlen. Bis sich neue Wege zur erfolgreichen Früherkennung von CCC auftun, bleiben wohl regelmäßige sonographische Kontrollen (z.B. alle 6 Monate) in Kombination mit jährlichen MRT-Untersuchungen und seriellen CA19-9-Bestimmungen ein gangbarer und pragmatischer Zugang, auch wenn bisher die zu erbringende Evidenz für die Wirksamkeit eines solchen Vorgehens hinsichtlich des Beweises eines besseren Überlebens im Falle einer CCC-Entwicklung unzureichend ist.

## Abkürzungen

AIH: Autoimmunhepatitis
ALT: Alanin-Aminotransferase
ANA: Antinukleäre Antikörper
ANCA: Antineutrophile zytoplasmatische Antikörper
AP: Alkalische Phosphatase
ASMA: Antikörper gegen glatte Muskulatur
AST: Aspartat-Aminotransferase
CA19‑9: „Carbohydrate antigen 19-9“
CCC: Cholangiozelluläres Karzinom
CED: Chronisch-entzündliche Darmerkrankung
CPG: Richtlinien für klinische Praxis („clinical practice guidelines“)
CRC: Kolorektales Karzinom
DHC: Ductus hepatocholedochus
ELF: „Enhanced liver fibrosis“
ELTR: Europäisches Lebertransplantationsregister
ERCP: Endoskopische retrograde Cholangiopankreatikographie
HCC: Hepatozelluläres Karzinom
IAC: IgG4-assoziierte Cholangitis
LTx: Lebertransplantation
MRCP: Magnetresonanzcholangiopankreatikographie
NIT: Nichtinvasiver Fibrosetest
PSC: Primär sklerosierende Cholangitis
UDCA: Ursodesoxycholsäure
ULN: Obere Normgrenze („upper limit of normal“)
